# The Dental Pulp

**Published:** 1891-04

**Authors:** W. Xavier Sudduth

**Affiliations:** Professor of Pathology and Oral Surgery in the University of Minnesota.


ARTICLE V.

### THE DENTAL PULP.

BY W. XAVIER SUDDUTH, A. M., M. D., D. D. S.,

Professor of Pathology and Oral Surgery in the University of Minnesota.

Objection has been raised against the use of the term dental pulp, some holding that the formative organ of the dentine should be called the dental nerve, as it is even now designated by the laity, because of the fact that when in an inflamed condition it is extremely sensitive.

Considered from the stand-point of histology and compared with other portions of the body, we find that it is much less highly organized and presents few of the special characteristics which entitle it to classification as nervous tissue. The greater portion of the mature pulp is composed of ordinary connective-tissue elements, being made up of filiform and fusiform cells, presenting no regularity in arrangement, but crossing and anastomosing in all directions.

Upon the surface of the pulp, forming part of it, is a layer of cells termed odontoblasts. They are developed from the ordinary embryonic connective tissue cells, but are specially endowed with the function of secreting the dentine. These cells are possessed of one or more fibrous prolongations which extend into the tubuli of the dentine. To these fibers of the odontoblast has been given the name of dentinal fibers because of their location.

The pulp is a very vascular organ. Its blood-supply consists of a few vessels of considerable size and numerous capillaries, which ramify throughout the entire organ.

It is also well supplied with nerve-elements. These consist of medullated and non-medullated nerve-fibers which terminate in the odontoblastic layer. They do not, however, present the same fineness of subdivision as is found in the terminal nerve fibers of the cornea.

Some writers hold that the sensitivity of inflamed dentine in decay indicates the presence of nerve-fibers in the dentinal tubuli. Such is not necessarily an absolute deduction, as an inherent property of protoplasm is the power of conducting sensation, which latter is not confined to nerve-fiber alone. It is also well known that in inflammation this property is highly exalted. The term sensitive dentine is a misnomer, as the sensation is confined to the dentinal fibrillæ, and the degree of sensitivity is in the inverse ratio to the attenuation of the fiber; hence the most sensitive point is found at the union of the enamel and dentine, where the fibers reach the finest sub-division. The more delicate the filament the more exalted its power of transmitting sensation, and inflamed dentine does not present any distinctive features not exemplified in other portions of the body under similar inflammatory conditions.

The most common conditions of inflammation that dentists come in contact with are pulpitis and periodontitis. They are not, as a rule, called upon to treat inflammations in other portions of the body, consequently are but little acquainted with inflammation in its general aspect. There are several places where inflammation is even more intense and painful than it is in the pulp. The eye is a most notable example, also the ear and the organs of generation, not to speak of the central nervous system.

Periodontitis is allied to inflammation in the periosteum, and differs in no whit from such processes wherever found in connection with the osseous system, and is always very painful. It appears to be the tendency of dentists to magnify the intensity of the conditions they are called upon to treat, largely, as we have intimated, because of their limited acquaintance with the processes of inflam mation in general, while the tendency among physicians is to minimize the conditions, because of their lack of knowledge of the special conditions found in the mouth.

It is not our intention to displace the dental pulp from the list of sensitive tissues when inflamed, because it does not differ from other tissues under similar conditions, but to compare it with other tissues in order to impress upon the mind the need of greater care in manipulation. Dentists, as a rule, when judged from the stand-point of delicacy of touch and carefulness in manipulation, make a very poor showing when compared with ophthalmologists.

An indifference to expressions of pain seems to grow upon some dental practitioners as practice increases, and they find themselves growing less appreciative of the niceties of practice which gained for them their reputation as careful operators. If the above stricture is true, and we firmly believe it is, then the reputation of the dental pulp to be the most sensitive organ of the body is largely due to our carelessness as operators.

Before dentistry was known, the favorite expression for a condition of extreme sensitivity was "as tender as the apple of the eye," but now, thanks to an extended acquaintance with the operating chair, the more modern phrase, "as tender as the nerve of a tooth," has been introduced. This comparison is not intended to be invidious, nor to build up one specialty at the expense of another. It is well, however, at times, to see ourselves as others see us. Let us see to it in the future that the incubus is removed from our shoulders, and the "apple of the eye" restored to its former position as the most fitting term of comparison for extreme sensitivity.—*Cosmos.*

